# Removal of a Distally Broken Cannulated Femur Intramedullary Nail: A Novel Technique From a Level 1 Trauma Center

**DOI:** 10.1155/cro/6220126

**Published:** 2025-02-25

**Authors:** Jeffrey Lucas Hii, Christopher J. Fang, Samantha L. Evans, Matthew Schuch, Erik N. Kubiak

**Affiliations:** University of Nevada Las Vegas Department of Orthopaedics, University of Nevada, Las Vegas, Nevada, USA

## Abstract

This case report from a Level 1 trauma center describes a novel surgical technique to remove a cannulated intramedullary nail, broken at the distal aspect, from the femur. We present a 40-year-old male who sustained a hardware failure, breaking his medullary nail at the distal aspect 7 weeks postoperatively while performing water aerobics. The broken implant was successfully extracted without complication, and a subsequent nail was exchanged. A benefit of this technique is avoiding a femoral osteotomy, which may prove useful for the unique and difficult case of distally broken nails.

## 1. Introduction

Catastrophic failure of intramedullary nail hardware poses a difficult challenge for management. Further, the extraction of a distal fragment of a broken nail represents a particularly technical dilemma. Various techniques have been described to extract a broken distal nail with variable success; however, there has yet to emerge a technique that can be used as a gold standard. In this paper, we propose and present a relatively cost-effective and minimally invasive technique from a Level 1 trauma center in the United States to extract a distally broken femoral nail.

## 2. Case Report and Operative Technique

**Figure 1:**
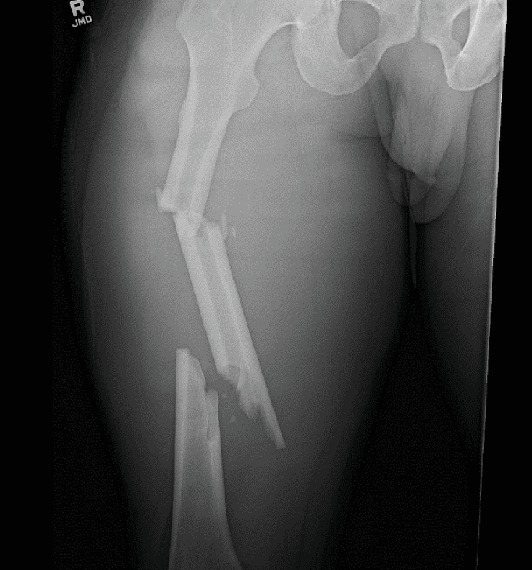
Segmental femur fracture.

**Figure 2:**
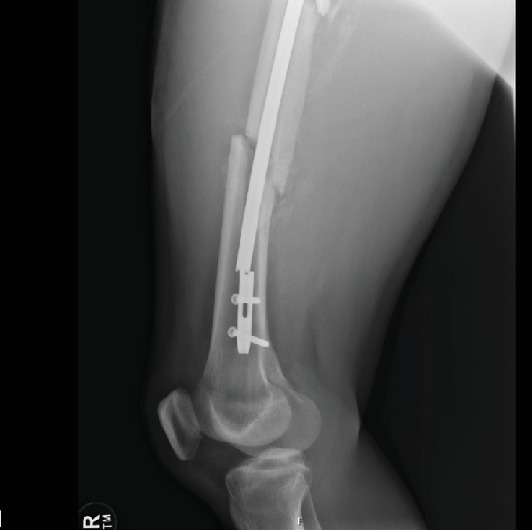
Image of broken femur nail.

A 40-year-old male patient with no significant past medical history was consulted by our service after sustaining a closed, segmental femur fracture following a motorcycle collision (Figure 1). A Synthes TFN (trochanteric fixation femoral nail) 420 cm in length by 10 mm in diameter with a 125° angle proximally was inserted in standard fashion (Synthes, Paoli, PA, United States). The patient was discharged from the hospital and seen at the 2- and 4-week follow-up appointments without any complications. At the 7-week mark, the patient presented to the clinic for new-onset distal thigh pain for which plain films revealed a hardware breakage of his distal nail through the interlocking screw hole (Figure 2). Subsequently, the patient was indicated for an extraction of hardware and nail exchange.

**Figure 3:**
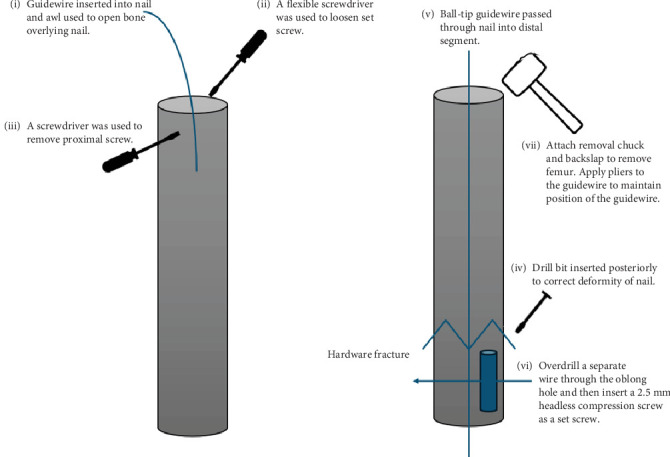
Summarized technique guide for the novel technique of removing a distally broken intramedullary nail.

**Figure 4:**
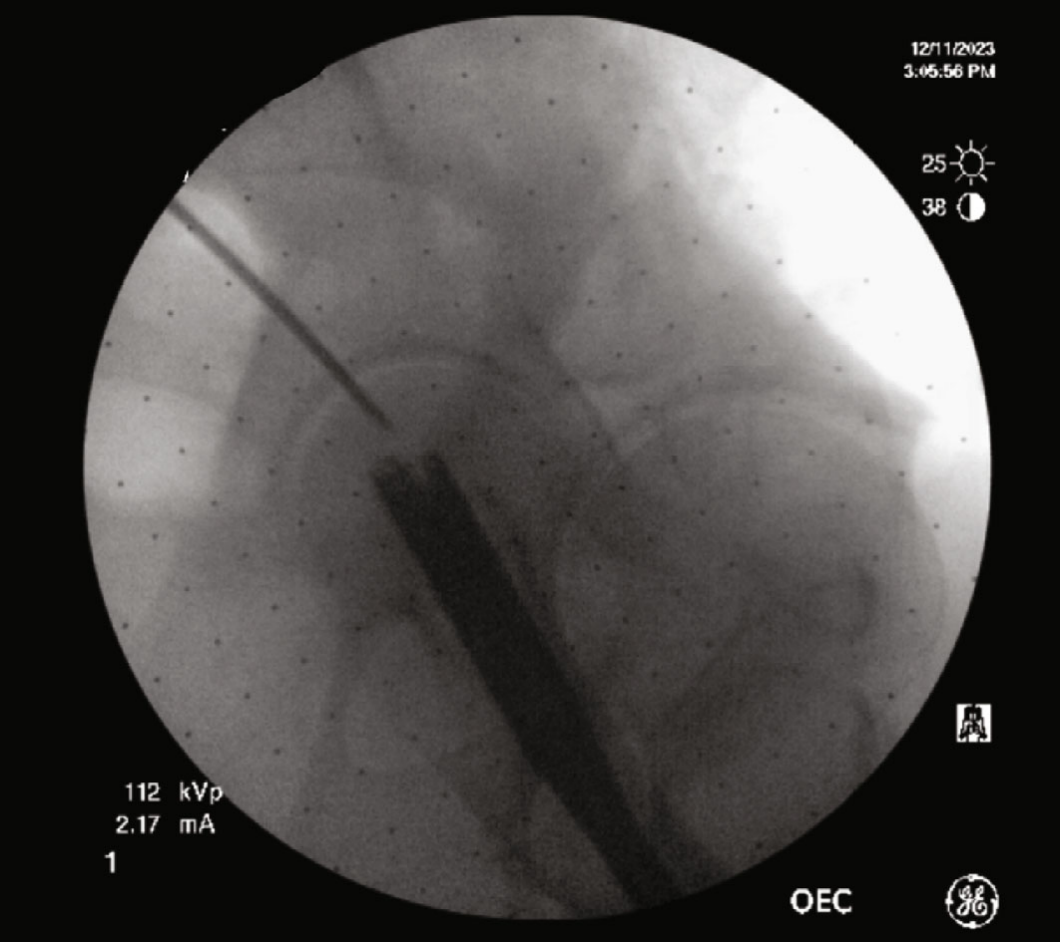
Starting point for the novel technique of removal of femur nail.

**Figure 5:**
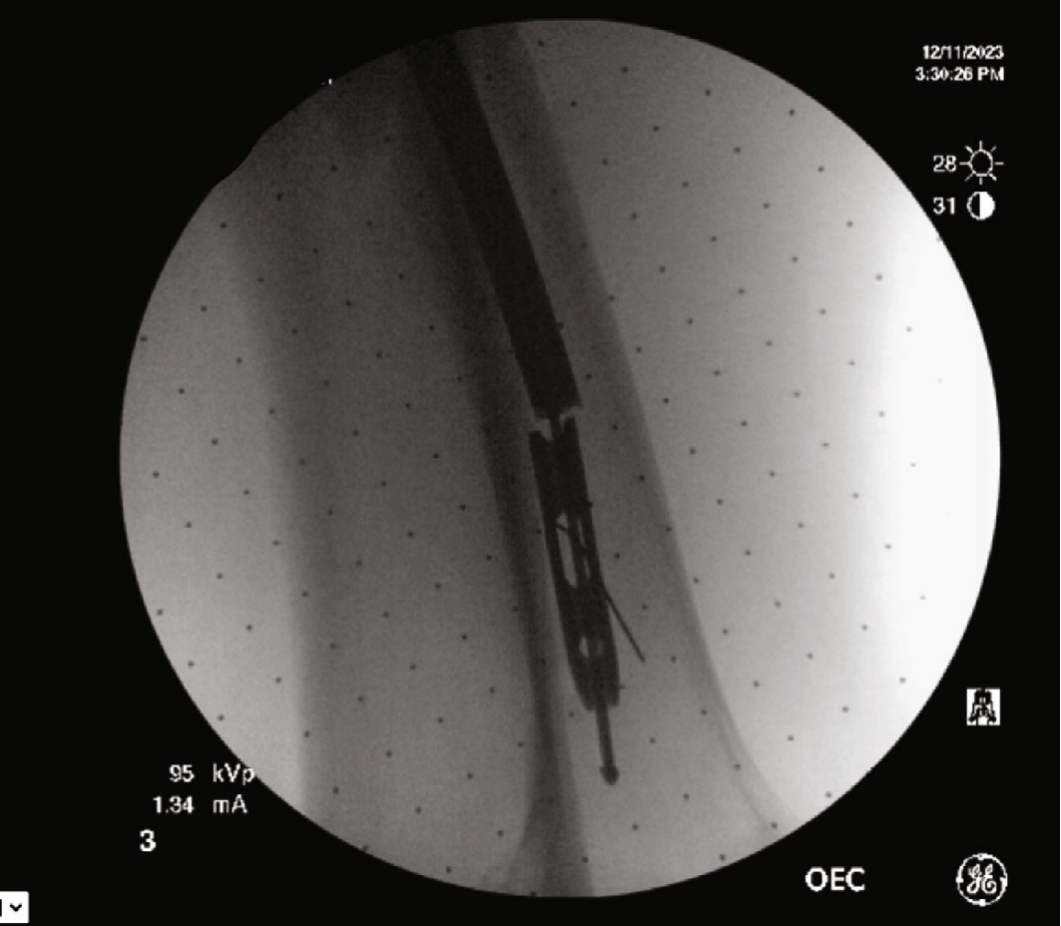
Image of the advancement of ball tip guide wire.

**Figure 6:**
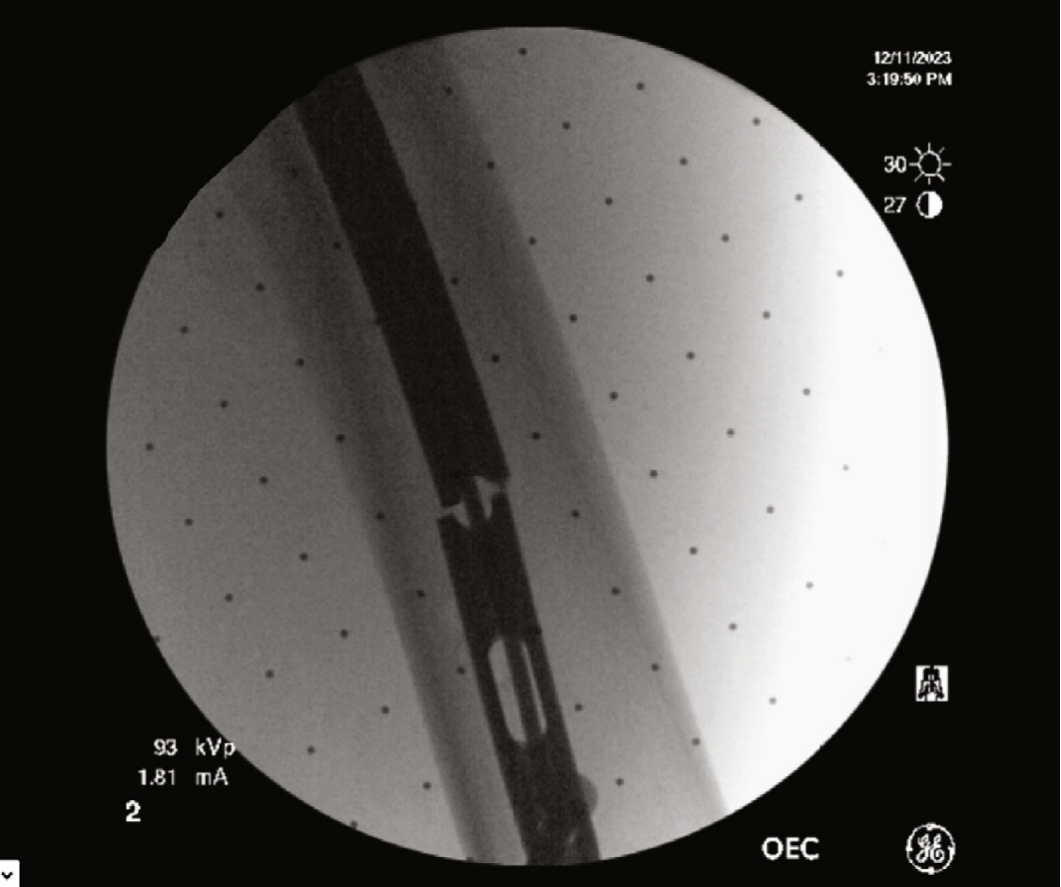
Insertion of the drill bit into the femur nail.

**Figure 7:**
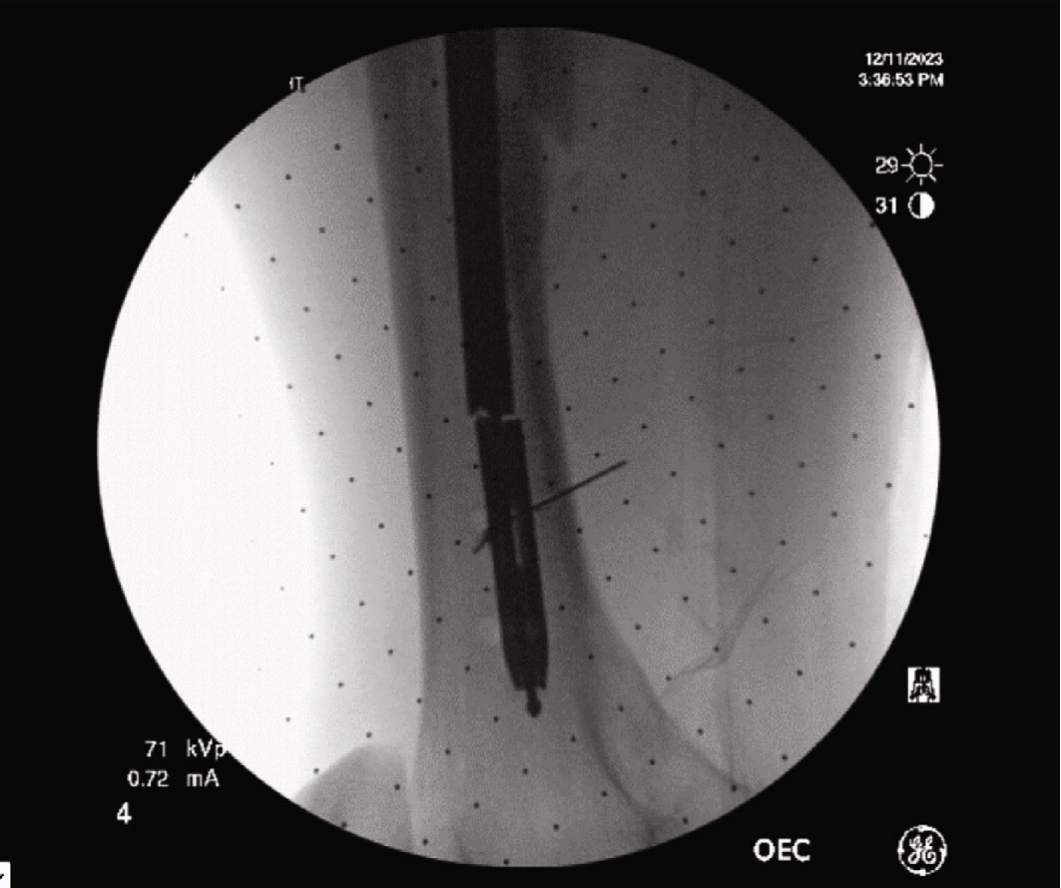
Image of wire and headless compression screw.

**Figure 8:**
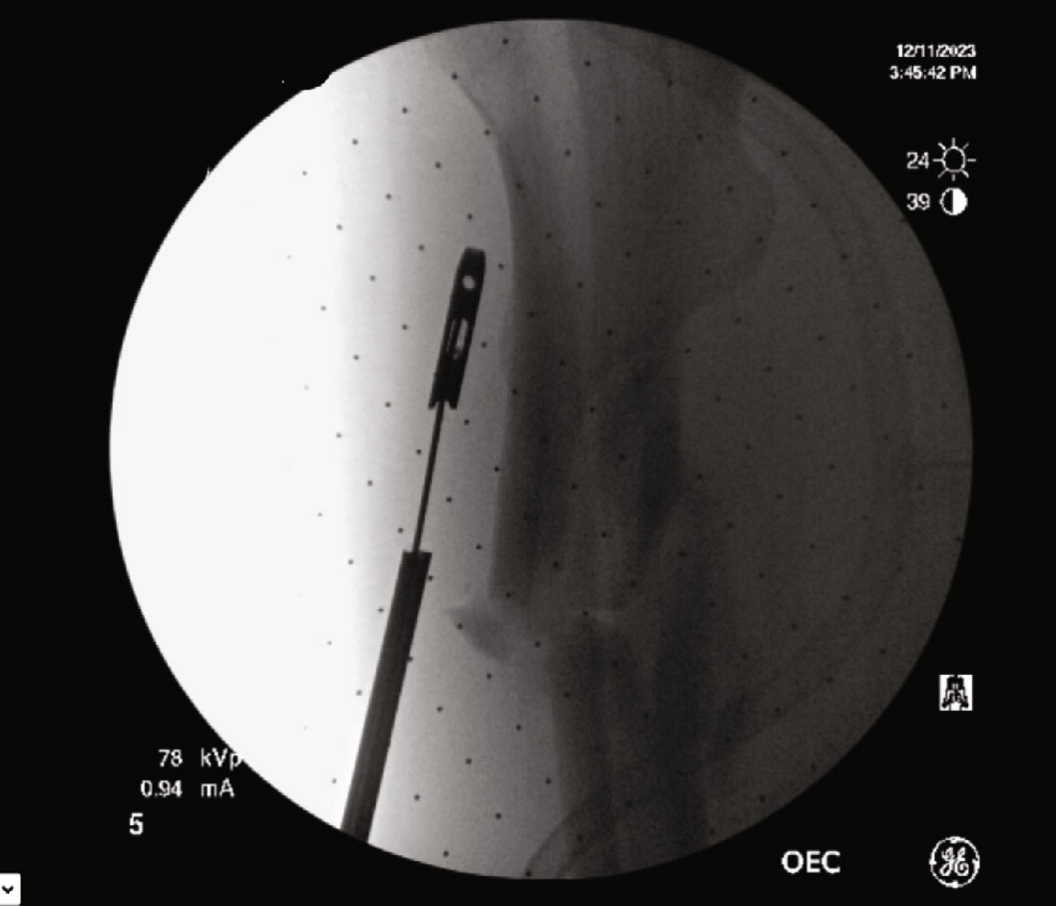
Removal of broken femur nail.

The patient was placed in the lateral decubitus position on an OSI (MIZUHO OSI Modular Table System) flattop bed. The following describes our novel technique to remove the broken intramedullary nail (Figure 3). Our technique removes both the proximal and distal aspects of the broken nail in one step. This eliminates the need for distal aspect extraction techniques such as the stacking technique or using a distractor hook. This technique is also performed closed in order to avoid violating the surrounding soft tissue envelope seen with open techniques. Utilizing the patient's previous incision, the starting point was made posterior to the greater trochanter of the femur (Figure 4). The previous nail was localized with a guidewire, and an awl was used to open the bone overlying the previous nail. The flexible screwdriver was then used to loosen the set screw. On anterior to posterior and lateral imaging, the cephalad screw was localized with the driver, and a small stab incision was made to remove the cephalad screw without complication. The ball-tip guidewire was passed through the nail, crossing the hardware fracture site into the distal segment (Figure 5). To achieve this, a drill bit was inserted into the posterior femur to block and translate the nail forward, correcting the apex posterior deformity of the broken nail (Figure 6). The ball-tip guidewire was secured to the distal nail fragment by overdrilling a separate wire through the oblong hole of the distal broken segment of the nail and inserting a 2.5–10 mm headless compression screw through the oblong distal interlocking hole to act as a set screw (Figure 7). The removal chuck was attached to the proximal guidewire, and the guidewire-nail construct was backslapped and removed from the femur without complication (Figure 8). Of note, it is important to place pliers on the guidewire adjacent to the nail extraction device and to back slap the nail while maintaining the position of the pliers on the guidewire so that the interference between the headless compression screw, the guidewire, and the nail is maintained while removing the nail.

## 3. Result

The broken nail was successfully removed, and a subsequent nail was exchanged without complication. This technique avoided opening and violating the soft tissue and used the previous proximal starting point, avoiding violation of the knee or any osteotomies.

## 4. Discussion

Distal hardware breakage of femur nails poses a particularly difficult challenge with serious potential complications for the orthopaedic surgeon. No gold standard technique exists for this complication, but various techniques have been described in the literature. These include strategies such as interference fit guide wires, hooks, stacking technique, double plain, and ball-tip guidewires (Table 1; Refs. [1–8]).

Each comes with its own profile of advantages and disadvantages. These disadvantages include knee violation, cortical windows, special and expensive equipment, and limited use for nails only larger than 10 mm in diameter. There are risks of our novel technique, such as jamming or incarceration during the backslap portion of the case; however, these are also risks with other removal techniques. If this complication occurs, the option would be to switch to more invasive techniques that are described in the more conventional nail removal techniques, such as an osteotomy. Our described technique has the benefits of not needing any special or costly equipment and is relatively minimally invasive without the need for any significant cost of tissue or cortical violation.

Likely, the ideal technique would be one tailored to the specific patient situation, and having an assortment of different techniques at the surgeon's disposal is best. Therefore, we submit our technique as another technique that may be of benefit in a markedly difficult situation.

## 5. Conclusion

We present a relatively cost-effective and minimally invasive technique for the extraction of broken nails with a technically challenging distal fragment. This technique is free from the constraints of needing special extraction equipment and avoids complications seen from open, osteotomy-reliant, or knee-violating techniques.

## Figures and Tables

**Table 1:** Broken nail removal techniques.

**Authors**	**Nail breakage**	**Technique**	**Journal**	**Year**	**Advantage**	**Disadvantage**
Sakellariou et al.	Mid-nail	Lateral longitudinal femoral osteotomy	*CRO*	2011	Bent/distorted nails	Osteotomy
Riansuwan, Tantigate, and Mahaisavariya	Distal 1/3	Harrington rod modified for retrograde impaction	*CRO*	2013	Avoids slippage as a cause of technical failure	Knee violation
Abdelgawad and Kanlic	Tibial nail	Hook captured in the medulla by a flexible nail introduced from the locking hole. Hook with stacking from the locking hole	*CRO*	2013	Small diameter nails	Limited use
Iqbal et al.	Mid-nail	Twisted plain and ball-tipped guidewires	*SICOT-J*	2021	Union or hypertrophic nonunion, no special equipment	Small diameter nails < 10 mm
Metikala and Mohammed	Distal 1/3	Ball tip guidewire through the knee, proximal extraction	*Indian J Orthop*	2011	Distal breakage, no special equipment	Knee violation
Zhao and Slater	Distal 1/3	Antegrade guide wire passed through the nail and pulled out of the cortical window with a push from a flexible reamer	*Injury*	2017	No special equipment, no disturbance of fracture site	Cortical window. Large or distal fragments
Pongsamakthai, Apivatthakakul, and Sangkomkamhang	Mid-nail	T reamer impacted into nail fragment with proximal extraction	*JCOT*	2016	No special equipment	Appropriate T reamer
Mazzini, Martin, and Erasun	Distal 1/3	Pull out with cement rongeur and extraction hook	*Strategies Trauma Limb Reconst*	2009	Unusual pattern breakage	Special equipment, disturbance of fracture site
